# Genomic Analysis Unravels Reduced Inorganic Sulfur Compound Oxidation of Heterotrophic Acidophilic* Acidicaldus* sp. Strain DX-1

**DOI:** 10.1155/2016/8137012

**Published:** 2016-04-28

**Authors:** Yuanyuan Liu, Hongying Yang, Xian Zhang, Yunhua Xiao, Xue Guo, Xueduan Liu

**Affiliations:** ^1^School of Materials and Metallurgy, Northeastern University, Shenyang, China; ^2^CNMC Luanshya Copper Mines Plc. (CLM), Luanshya, Zambia; ^3^School of Minerals Processing and Bioengineering, Central South University, Changsha, China

## Abstract

Although reduced inorganic sulfur compound (RISC) oxidation in many chemolithoautotrophic sulfur oxidizers has been investigated in recent years, there is little information about RISC oxidation in heterotrophic acidophiles. In this study,* Acidicaldus* sp. strain DX-1, a heterotrophic sulfur-oxidizing acidophile, was isolated. Its genome was sequenced and then used for comparative genomics. Furthermore, real-time quantitative PCR was performed to identify the expression of genes involved in the RISC oxidation. Gene encoding thiosulfate: quinone oxidoreductase was present in* Acidicaldus* sp. strain DX-1, while no candidate genes with significant similarity to tetrathionate hydrolase were found. Additionally, there were genes encoding heterodisulfide reductase complex, which was proposed to play a crucial role in oxidizing cytoplasmic sulfur. Like many heterotrophic sulfur oxidizers,* Acidicaldus* sp. strain DX-1 had no genes encoding enzymes essential for the direct oxidation of sulfite. An indirect oxidation of sulfite via adenosine-5′-phosphosulfate was proposed in* Acidicaldus *strain DX-1. However, compared to other closely related bacteria* Acidiphilium cryptum* and* Acidiphilium multivorum*, which harbored the genes encoding Sox system, almost all of these genes were not detected in* Acidicaldus* sp. strain DX-1. This study might provide some references for the future study of RISC oxidation in heterotrophic sulfur-oxidizing acidophiles.

## 1. Introduction

Due to the evolutionary importance and applied perspectives of extreme acidophiles (optimal growth at or below pH 3.0), researchers have paid much attention to these microorganisms [1–3]. Extreme acidophiles are widely used in the bioprocessing of minerals and bioremediation of acidic and metal-enriched water [4–6]. Many chemolithoautotrophic acidophiles, such as* Leptospirillum* spp. and* Sulfolobus metallicus*, can accelerate the dissolution of sulfide minerals by oxidizing ferrous iron or reduced inorganic sulfur compound (RISC) [7]. In addition to obligate autotrophs, many heterotrophic and mixotrophic acidophiles also play key roles in iron and sulfur cycling, such as* Ferrimicrobium acidiphilum, Sulfobacillus *spp.,* Thermoplasma* sp., and* Acidiphilium *spp. [7–12].

Species from the* Acidicaldus* genus are moderately thermophilic and obligate heterotrophic acidophiles within the Alphaproteobacteria class. The type species* Acidicaldus organivorans* Y008, which was isolated from a geothermal site in Yellowstone National Park, is a member of facultatively anaerobes and can grow by ferric iron respiration in the anaerobic environment [13]. The closest phylogenetic relatives of* Ac. organivorans *Y008 are acidophilic heterotrophs, including* Acidiphilium *[14],* Acidisphaera *[15], and* Acetobacter *[16]. Previous studies showed that* Acidicaldus organivorus* (the synonym of* Ac*.* organivorans*) harbored some unique physiological characteristics, which differed from other acidophilic heterotrophs. Compared to* Acidisphaera rubrifaciens*, which grows at pH 4.5–5.0 and optimal temperature 30–35°C,* Acidicaldus organivorus* requires a lower pH (2.5–3.0) and higher temperature (optima 50–55°C).* Ac. organivorus* can oxidize elemental sulfur to sulfate, though it cannot perform autotrophic growth on sulfur in organic-free media. Additionally,* Ac. organivorus* can catalyze the dissimilatory reduction of ferric iron, whereas the type strain of* As. rubrifaciens* does not grow by ferric iron respiration in the absence of oxygen [13].

Although knowledge in the last decade has greatly advanced our understanding on the acidophilic bacteria, it is scarce to attempt to study the physiology and genetics of the heterotrophic sulfur-oxidizing acidophiles. Compared to autotrophic sulfur-oxidizing bacteria, metabolisms of these heterotrophs are more versatile [17]. They can not only use organic matters as energy sources but also obtain energy derived from the oxidation of elemental sulfur or RISC. These traits can confer the heterotrophic sulfur-oxidizing bacteria dual contributions to microbial communities in bioleaching systems: (i) while organic matters inhibit the growth of some facultative autotrophic bacteria [18, 19], heterotrophs which can consume the organic matters in the microbial communities might relieve the inhibiting effect of organic matters on the autotrophs; (ii) RISC oxidation would accelerate dissolution of acid soluble metal sulfides. In this study, we chose a strain, isolated from Dexing Copper Mine (Jiangxi, China), to explore its physiologic and genetic properties. The genome sequence of this strain was acquired using the whole genome sequencing and comparative genomics was then performed, aiming to propose its putative RISC oxidation model.

## 2. Materials and Methods

### 2.1. Isolation and Cultivation of Bacteria

Samples from the surface of effusion pool were inoculated in 9K liquid media [20] supplemented with 5 g/L elemental sulfur, 4.5% (w/v) ferrous sulfate, and 0.02% yeast extract (Oxoid, UK). Strains were purified by repeated single colony (in triplicate) isolation on 9K plates solidified with 0.02% yeast extract and grown at 45°C in 9K liquid media, pH 3.0, with 0.02% (w/v) yeast extract and 10 mM glucose. All procedures were operated aseptically.

### 2.2. DNA Extraction, 16S rRNA Gene Amplification, and Phylogenetic Analysis

At later exponential phase (generally 4th day), strain DX-1 was harvested by centrifugation (12,000 g for 10 min at 4°C). Genomic DNA was extracted from the pelleted cells using TIANamp Bacteria DNA Kit (Tiangen Biotech, China) according to the manufacturer's instruction and finally was resuspended in TE buffer. 16S rRNA gene sequence was PCR amplified from the genomic DNA extracts of strain DX-1. Amplification was performed in 50 *μ*L reaction mixtures containing 1 *μ*L of DNA extracts, 1 *μ*L each of 10 mM forward and reverse primers, 25 *μ*L of universal Taq PCR Master Mix (Tiangen Biotech, China), and 22 *μ*L of deionized water. The PCR conditions for amplification were as follows: 94°C for 4 min, then 32 cycles of 94°C for 30 s, 55°C for 30 s, and 72°C for 45 s, followed by a final extension at 72°C for 10 min. PCR product of 16S rRNA gene was purified directly with the QIAquick PCR Purification Kit (Qiagen, Germany) and sequenced.

16S rRNA gene sequence of strain DX-1 was aligned with other acidophilic Alphaproteobacteria sequences from the GenBank database using the CLUSTAL program [21]. This alignment was used to make a distance matrix. Phylogenetic trees were constructed using the neighbor joining method by Molecular Evolutionary Genetics Analysis 5.2 (MEGA, version 5.2) [22]. Bootstrap analysis was carried out on 1000 replicate input data sets.

### 2.3. Genome Sequencing, Assembly, and Annotation

Genomic library construction, sequencing, and assembly were performed according to previous methods [23]. Given the high GC content of genome of* Acidicaldus *spp., more than 600 Mb pair-ends reads with a depth of over 200-fold coverage were obtained. After genome assembly, 375 scaffolds were acquired using SOAPdenovo package [24]. The completeness (95.34%) of strain DX-1 genome was estimated using the CheckM [25]. Coding sequences (CDSs) were then predicted with the ORF finders Glimmer [26]. All CDSs were annotated by comparison with the public available databases nonredundant NCBI [27] and KEGG [28] using the annotation software BLAST [29]. The unassigned CDSs were further annotated using the HMMPfam program [30]. And the hidden Markov models for the protein domains were obtained from the Pfam database [31]. The software programs tRNAscan-SE and RNAmmer were used for the identifications of tRNA and rRNA, respectively [32, 33].

### 2.4. Comparative Genomics

The genomes of* Ap. cryptum* JF-5 and* Ap. multivorum* AIU301 were retrieved from the NCBI database. Orthologs between* Acidicaldus* strain DX-1,* Ap*.* cryptum* JF-5, and* Ap*.* multivorum* AIU301 were detected via an all-versus-all reciprocal BLASTP search against their own protein sets, respectively [29]. The best sequence similarities were obtained using two cut-off values: *E*-value ≤ 1*e* − 05 and minimal coverage by local alignment = 70%.

### 2.5. RNA Extraction and Real-Time Quantitative PCR Analysis

To investigate the transcript profiles of genes associated with RISC oxidation,* Acidicaldus* sp. strain DX-1 was cultured at 45°C in 9K liquid media, pH 3.0 with 0.02% (w/v) yeast extract and 10 mM glucose. And the additional 1% (w/v) elemental sulfur (S^0^) was added in another group. Microbial cells were harvested at mid-exponential growth phase according to bacterial growth curve. Total RNA was extracted using TRIzol reagent (Invitrogen, Carlsbad, USA), treated with RNase-free DNase I (Qiagen, Valencia, USA) and purified with RNeasy Kit (Qiagen, Valencia, USA). Subsequently, single-stranded cDNA was synthesized with ReverTra Ace qPCR RT Kit (Toyobo, Japan), according to the manufacturer's protocol. The cDNA was stored at −80°C, which was used for further RT-qPCR analysis. All experiments under the same conditions were performed in triplicate. The gene expression level with fold change ≥ 1 were upregulated and otherwise downregulated.

Specific primers targeting selected genes putatively involved in RISC oxidation were designed for real-time PCR analysis (Table 1). The real-time PCR amplification was performed with iCycler iQ Real-Time PCR detection system (Bio-Rad Laboratories Inc., Hercules, USA). 25 *μ*L reaction mixtures contained 12.5 *μ*L SYBR Green Real-Time PCR Master Mix (Toyobo Co., Ltd., Osaka, Japan), 0.5 *μ*L single-stranded cDNA, 1 *μ*L each of 10 *μ*M forward and reverse primers, and 10 *μ*L deionized water. The specific amplification protocol was as follows: 95°C for 5 min, 40 cycles of 95°C for 20 s, 55°C for 15 s, 72°C for 15 s, and a final incubation of 72°C for 10 min. The expression of each gene was determined from triplicate reactions in a single real-time PCR amplification. To standardize the quantification of the selected target genes, 16S rRNA and glyceraldehyde 3-phosphate dehydrogenase (*gapdh*) were used as transcription controls to regulate the random and systematic errors. The expressions of selected genes in the medium with S^0^ and without S^0^ were calculated for further analysis.

### 2.6. Nucleotide Sequence Accession Number

The draft genome sequence of* Acidicaldus* strain DX-1 has been deposited at DDBJ/EMBL/GenBank under accession number JPYW00000000.

## 3. Results and Discussion

### 3.1. Isolation and Phylogenetic Analysis

**Figure 1:**
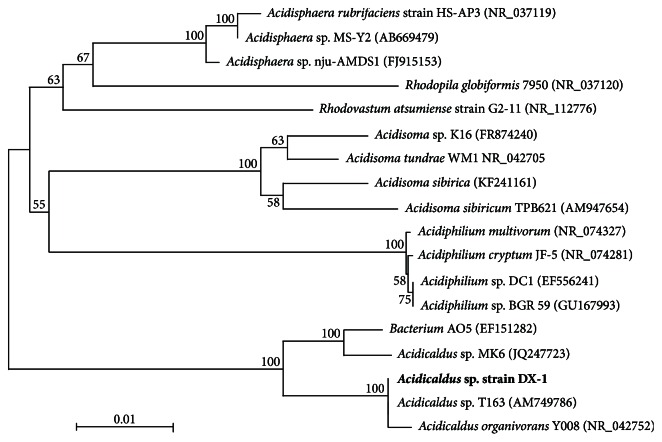
Phylogenetic tree of 16S rRNA genes showing the relationship of strain DX-1 (in* bold*) to other acidophilic *α*-Proteobacteria. Phylogenetic tree was constructed based on an alignment of 1283 bp using the neighbor joining method. The scale bar represents 0.01 nucleotide substitutions per 100. And the database accession numbers of the gene sequences used are given in parentheses.* Acidisphaera rubrifaciens* HS-AP3 was used to root the tree.

Several strains (named DX-1, DX-5, DX-20, and DX-22) from Dexing Copper Mine sampling sites, which showed similar colony and cellular morphologies, were isolated on solid medium with 0.02% yeast extract. All of these strains grew as small, white, round to convex-shaped colonies. Considering that 16S rRNA sequence analysis showed 100% sequence identities, only one (strain DX-1) was selected for further analysis. The rooted phylogenetic tree indicated that 16S rRNA gene of strain DX-1 was assigned into a phyletic cluster with* Acidicaldus *spp. (Figure 1). Furthermore, 16S rRNA gene of strain DX-1 shared 99% sequence identity with that of type strain* Acidicaldus organivorans *Y008 [13], indicating that strain DX-1 belongs to* Acidicaldus* species.

### 3.2. Genome Assembly and Annotation

The draft genome sequence of* Acidicaldus* sp. strain DX-1 contained 2,990,377 bp with GC content of 68.48% distributed in 376 scaffolds, which ranged from 1,000 bp to 67,370 bp. Given the high sequence coverage (200-fold), it was likely to identify most genes in the draft genome of* Acidicaldus* sp. strain DX-1. Results showed that genome of* Acidicaldus* sp. strain DX-1 harbored 3,259 predicted coding sequences (CDSs), which represented 89.83% of the genome, 1 rRNA operon, and 43 tRNA genes (Table 2). The number of CDSs in this strain was less than that in* Acidiphilium multivorum* AIU301 but more than that in* Acidiphilium cryptum* JF-5 and* Acetobacter pasteurianus*.

### 3.3. Hydrogen Sulfide Oxidation

Phototrophic bacteria such as* Allochromatium vinosum* and some* Chlorobium* spp. can anaerobically oxidize hydrogen sulfide to sulfur via a flavocytochrome *c*-sulfide dehydrogenase [34]. In this process, cytochrome *c*_550_ is used as electron acceptor for energy production. However, flavocytochrome *c* in* A. vinosum* is not required for phototrophic growth with hydrogen sulfide. Therefore, sulfide: quinone reductase (SQR) is considered to be essential for growth of* A. vinosum* with hydrogen sulfide. In chemotrophic bacteria, such as some* Acidithiobacillus* spp., SQR is also responsible for the oxidation of hydrogen sulfide [35]. In* Acidicaldus* sp. strain DX-1, no candidate genes encoding flavocytochrome *c*-sulfide dehydrogenase were identified, while two genes encoding SQR were found (Table S1, see Supplementary Material available online at http://dx.doi.org/10.1155/2016/8137012). One putative SQR shared 64% amino acid similarity with that of* At. ferrooxidans* ATCC 53993, and the other shared 72% amino acid similarity with that of* Bradyrhizobium oligotrophicum* S58. Both of them had a small NADH binding domain within a larger FAD binding domain. These results suggested that SQR might be responsible for the oxidation of hydrogen sulfide in* Acidicaldus* sp. strain DX-1.

### 3.4. S_4_I Pathway

In some strains of the genus* Acidithiobacillus*, the membrane-bound thiosulfate: quinone oxidoreductase (TQO) and tetrathionate hydrolase (TetH) are responsible for the S_4_I pathway [36, 37]. In* At. ferrooxidans*, TQO which is constituted of subunits DoxA and DoxD catalyzes the conversion of thiosulfate to tetrathionate, and TetH catalyzes the hydrolysis of tetrathionate to generate sulfur, sulfate and thiosulfate. The consecutive reactions catalyzed by TetH and TQO promote the RISC oxidation in* At. ferrooxidans *[38]. One ortholog of* doxDA* encoding TQO was identified in* Acidicaldus* sp. strain DX-1, which shared 61% similarity with that in* At. ferrooxidans* (Table S1). Further analysis revealed that the putative TQO had conserved DoxD domain (Pfam: PF04173) and DoxA domain (Pfam: PF07680). Although previous experiments indicated that* Ac. organivorans *Y008 can grow on tetrathionate overlay medium [13], no candidate genes with significant similarity to TetH were found in the draft genome of* Acidicaldus* strain DX-1. Future analyses will be necessary to determine whether TQO indeed catalyzes thiosulfate to tetrathionate and TetH gene is on the portion of the missing draft genome of* Acidicaldus* sp. strain DX-1.

### 3.5. Sox Pathway

In the alphaproteobacterium* Paracoccus pantotrophus*, the periplasmic Sox system encoded by 15 genes [39] are comprised of four subunits, including SoxXA, SoxYZ, SoxB, and Sox(CD)_2_. Each Sox subunit directing its own function was elaborated in previous studies [40]. Both* Acidiphilium cryptum* and* Acidiphilium multivorum* have one gene cluster of* soxXYZABCD* to code for four periplasmic proteins. However, no candidate genes encoding Sox system were identified in the genome of* Acidicaldus* sp. strain DX-1. Considering the Sox system was absent, S_4_I pathway was probably the sole way of further oxidation of thiosulfate in periplasm.

### 3.6. Sulfur Oxidation

The cytoplasmic sulfur oxygenase reductase (SOR), which mediates biological oxidation of inorganic sulfur, can catalyze the disproportionation reaction of sulfur to generate sulfite, thiosulfate, and hydrogen sulfide [23, 38, 40–42]. Sulfur dioxygenase (SDO) is proposed to be an important elemental sulfur oxidation enzyme in the genus* Acidithiobacillus *[43], and the relevant gene of SDO and its enzyme activity have been identified in* At. caldus *[41]. Result showed that the genes* sor* and* sdo* were not identified in heterotroph* Acidicaldus *sp. strain DX-1. Another enzyme directing sulfur oxidation is the cytoplasmic heterodisulfide reductase complex [37], which catalyzes the oxidation of sulfane sulfate (RSSH) to produce sulfite. In* At. caldus*, RSSH is the product of thio proteins (RSH) with a sulfur atom in the catalysis of thiosulfate by rhodanese (TST) [41]. Results showed that one ortholog of TST was found in* Acidicaldus* sp. strain DX-1. Additionally, compared to other heterodisulfide reductase complex reported in previous studies [38, 44, 45], only two copies of* hdrB* gene, one cope of each HdrAC subunit, and* dsrE* gene were distributed in the draft genome of* Acidicaldus* sp. strain DX-1 (Table S1). We cannot confirm whether other components were present based on the draft genome alone. The putative HdrA subunit was flavoprotein that contained FAD binding site and conserved residues (C-X-G-X-R-D-X_6–8_-C-S-X_2_-C-C) for binding of Fe-S cluster, which shared 72% sequence similarity with that of* At. ferrooxidans*. Additionally, the putative HdrB having one typical cysteine-rich regions was distributed in the Hdr gene cluster, and the HdrC subunit having the 4Fe-4S ferredoxin iron-sulfur binding domain shared 74% and 71% sequence similarities with that of* At. ferrooxidans*, respectively.

### 3.7. Sulfite Oxidation

Another important step in the biological RISC oxidation was sulfite oxidation, which was involved in two different pathways. In these models, sulfite was (i) directly oxidized to sulfate, which was catalyzed by a molybdenum-containing sulfite: acceptor oxidoreductase or (ii) indirectly oxidized by the intermediate adenosine-5′-phosphosulfate (APS) [40]. For indirect sulfite oxidation, sulfite was catalyzed by APS reductase to produce APS and then oxidized to generate sulfate by sulfate adenylyltransferase (SAT). Most studies support the fact that the indirect sulfite oxidation exists in both* At. ferrooxidans *and* At. caldus*, which lack gene encoding APS reductase [36, 38]. In* Acidicaldus* sp. strain DX-1, genes coding for sulfite: acceptor oxidoreductase and APS reductase were not found. However, a putative gene encoding SAT, which has a conservative phosphoadenosine phosphosulfate (PAPS) reductase family domain, was identified in* Acidicaldus* sp. strain DX-1 (Table S1). Thus, indirect sulfite oxidation might also play an important role in* Acidicaldus *sp. strain DX-1.

### 3.8. Terminal Oxidases in RISC Oxidation

In chemolithoautotrophic sulfur-oxidizing bacteria, the RISC oxidation closely links with electron transfer through terminal oxidases. In* At. ferrooxidans*, electrons from RISC oxidation are transferred via the quinol pool (QH_2_) (i) either directly to terminal oxidase* bd* or* bo*_*3*_ to produce a proton gradient or indirectly through a* bc*_*1*_ complex and cytochrome *c* or a high potential iron-sulfur protein (HiPIP) to* aa*_*3*_ oxidase where the concentration of O_2_ is low or (ii) to NADH complex I to generate reducing power [38]. Genomic analysis showed that* Acidicaldus* sp. strain DX-1 had one copy of* bd* ubiquinol oxidase genes (*cydAB*), one gene cluster encoding* bc*_*1*_ complex, and a gene cluster encoding the* aa*_*3*_ oxidase (Table S2). Genes encoding 14 subunits of NADH complex I were also identified in its genome. However, no candidate genes encoding cytochrome* bo*_*3*_ ubiquinol oxidase were found. Therefore, a putative electron transfer chain in* Acidicaldus* sp. strain DX-1 was proposed: electrons from SQR, HDR, and TQO were transferred via the QH_2_ (i) either to* bd* oxidase or* bc*_*1*_ complex to produce the proton gradient or (ii) to NADH complex I to generate reducing power. Additionally, electrons in* bc*_*1*_ complex were probably transferred via cytochrome *c* to the* aa*_*3*_ oxidase, where the concentration of O_2_ is low, to produce the proton gradient.

### 3.9. Expression of Key Genes Involved in RISC Oxidation

**Figure 2:**
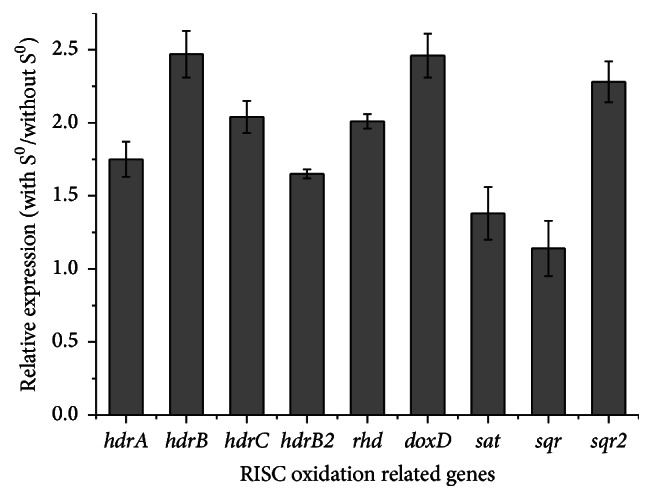
RT-qPCR results depicting the expression difference of targeted genes involved in RISC oxidation in* Acidicaldus* sp. strain DX-1 (in organic matter supplemented with sulfur (S^0^) medium or without sulfur). These genes used in this experiment (the encoding proteins and genomic loci are shown in parentheses) include* hdrA* (pyridine nucleotidedisulfide oxidoreductase; scaffold254: 1-941),* hdrB* (heterodisulfide reductase subunit B, homolog; scaffold254: 827-2329),* hdrC* (iron-sulfur cluster-binding protein; scaffold254: 2500-3246),* hdrB2* (heterodisulfide reductase subunit B, homolog; scaffold9: 22130-22789),* rhd* (rhodanese, thiosulfate sulfurtransferase; scaffold153: 15026-15202),* doxD* (thiosulfate: quinone oxidoreductase; scaffold68: 10659-11696),* sat* (sulfate adenylyltransferase; scaffold217: 14172-14891),* sqr* (sulfide quinone reductase; scaffold18: 14834-16117), and* sqr2* (sulfide quinone reductase; scaffold92: 4297-5430).

Genomic analysis provided evidence that most genes associated with RISC oxidation in* Acidicaldus *sp. strain DX-1 were similar to that in other acidophilic sulfur oxidizers. Furthermore, the expressions of relevant genes in both organic matter and organic matter supplemented with sulfur (S^0^) medium were validated by real-time quantitative PCR (Figure 2). Results indicated that all selected genes were upregulated in organic matter + S^0^ culture, suggesting that these encoding proteins are likely involved in the RISC oxidation in this strain. Genes encoding TST and three subunits of HdrABC complex for* Acidicaldus* strain DX-1 were significantly upregulated, while* hdrB2* gene has relatively lower expression. The conserved family domains of each HdrABC subunit and change of gene expression pattern strongly suggested that the HdrABC complex was concluded to oxidize cytoplasmic sulfur in* Acidicaldus* sp. strain DX-1. In particular, genes encoding HdrABC complex were not found in the closely related bacteria* Ap. cryptum* JF-5 and* Ap. multivorum *AIU301, which might be the reason why many strains of* Acidiphilium *spp. could not directly oxidize sulfur. Additionally, compared with* sqr* gene,* sqr2* gene was significantly upregulated, indicating its importance to metabolize the periplasmic sulfide which might be generated from other unknown metabolic pathways.

## 4. Conclusions

**Figure 3:**
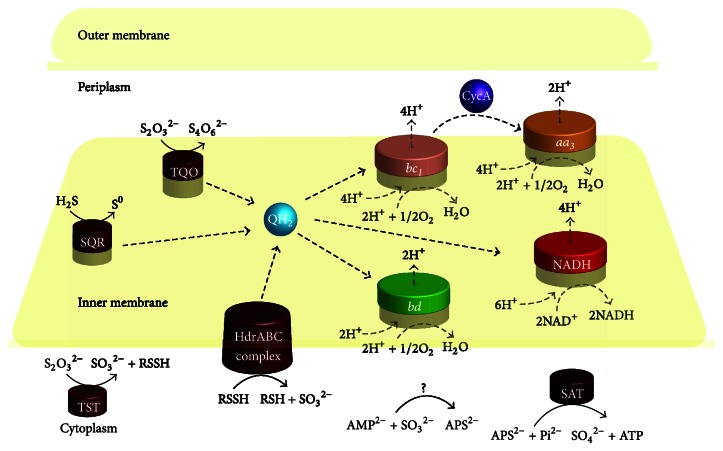
Genome-based model of RISC oxidation in* Acidicaldus* sp. strain DX-1. The figure was adapted from previous sulfur oxidation models [23, 38]. RISC oxidation involves various enzymes and a number of electron carriers. The solid lines denote the oxidation of RISC catalyzed by various enzymes, and the dashed lines denote the direction of electron transfer. SQR, sulfide quinone reductase; TQO, thiosulfate quinone oxidoreductase; TST, rhodanese; HdrABC, heterodisulfide reductase complex; SAT, sulfate adenylyltransferase;* bd*, terminal oxidase* bd*;* bc*_*1*_, terminal oxidase* bc*_*1*_ complex; NADH, NADH complex I;* aa*_*3*_, cytochrome *c* oxidase* aa*_*3*_-type; QH_2_, quinol pool; and CycA, cytochrome* c*.

In acidophilic microbial communities, heterotrophic sulfur-oxidizing bacteria have versatile functions, which not only can utilize numerous organic matters as energy sources but can also acquire energy from the oxidation of RISC. In this study, we isolated a heterotrophic sulfur-oxidizing bacterium,* Acidicaldus* sp. strain DX-1, from the effusion of mill tailings in Dexing Copper Mine. Comparative genomics and RT-qPCR analysis revealed that several genes associated with RISC oxidation in* Acidicaldus *sp. strain DX-1 were similar to other acidophilic sulfur-oxidizing bacteria. Based on the aforementioned analyses, a model for RISC oxidation in* Acidicaldus* sp. strain DX-1 was hypothesized (Figure 3), which might provide new insights and guides for the RISC oxidation of heterotrophs in the future.

## Supplementary Material

Table S1: Vairous enzymes associated with RISC oxidation in the *Acidicaldus* sp. strain DX-1.
Table S2: Terminal oxidases involving in the reduced inorganic sulfur compound oxidation of *Acidicaldus* strain DX-1.

## Figures and Tables

**Table 1:** Primers used for real-time quantitative PCR in this study.

Targeted gene	Primers	Product size [bp]	Melting temp. [°C]	Primer sequence
*hdrA*	Fwd Rev	262	55	GCACCGGCTTCACCCATTTC AGCTGCTCGCGGATCTCCAT

*hdrB*	Fwd Rev	184	55	ACTCGACCTGGTATGATTGCTGC TTGCCGATCCACTGGCTCTT

*hdrC*	Fwd Rev	161	55	CACGGCGTGGGAGGTCAATC CGGCGAGCCAGTCTTCGTAAT

*hdrB2*	Fwd Rev	201	55	ACAACGAACACGCGAAAGAGG GACGGACGTACATGCAGCCATA

*rhd*	Fwd Rev	238	55	CCCAACGAGGGCAAGGACGG AGAGGCGGAGCAGCCACCAG

*doxD*	Fwd Rev	103	55	CAACTGGATGGCGCACAAA CGCATAGAGCAGCGGGAAA

*sat*	Fwd Rev	119	55	GACCTTCTCGAAAGCCAGAGC TTGCGGGCAAGCCAAACC

*sqr*	Fwd Rev	182	56	CGATTTCGGCGACTCGGGTGT GCTGGCGGAGTAGGAATTTCTCAT

*sqr2*	Fwd Rev	258	55	AAACCGGGTGCGCTTCGT TGCGGCTCCTTCGGATTGC

**Table 2:** General features of *Acidicaldus* sp. strain DX-1 in comparison with other heterotrophic acidophiles.

Organism	*Acidicaldus* sp. strain DX-1	*Acidiphilium cryptum* JF-5	*Acidiphilium multivorum *AIU301	*Acetobacter pasteurianus*
Isolated from	Acid effusion of mill tailing	Acidic Sediments	Pyritic acid mine drainage	Spontaneous cocoa bean heap fermentation
Temperature range	Moderate thermophilic (50~55°C)	Mesophilic (30~35°C)	Mesophilic (30–35°C)	Mesophilic (25–30°C)
pH range	2.5–3.0	3.0–3.5	3.0–3.5	5.4–6.3
Nutrition type	Heterotrophic	Heterotrophic	Heterotrophic	Heterotrophic
Oxygen requirement	Facultatively anaerobic	Facultatively anaerobic	Facultatively anaerobic	Obligately aerobic
Fe(III) reduction	Yes	Yes	Yes	No
Sulfur oxidation	Yes	No	No	No
Genome size (Mb)	2.99	3.39	3.75	2.91
GC content	68.48%	67.99%	67.58%	53.04%
Protein-coding genes	3259	3063	3448	2875
rRNA operon	1	2	2	5
Number of tRNAs	43	47	48	57

## References

[1] Dopson M., Johnson D. B. (2012). Biodiversity, metabolism and applications of acidophilic sulfur-metabolizing microorganisms. *Environmental Microbiology*.

[2] Baker-Austin C., Dopson M. (2007). Life in acid: pH homeostasis in acidophiles. *Trends in Microbiology*.

[3] Cárdenas J. P., Valdés J., Quatrini R., Duarte F., Holmes D. S. (2010). Lessons from the genomes of extremely acidophilic bacteria and archaea with special emphasis on bioleaching microorganisms. *Applied Microbiology and Biotechnology*.

[4] Johnson D. B., Dziurla M.-A., Kolmert Å., Hallberg K. B. (2002). The microbiology of acid mine drainage: genesis and biotreatment: review article. *South African Journal of Science*.

[5] Zhang X., Niu J., Liang Y., Liu X., Yin H. (2016). Metagenome-scale analysis yields insights into the structure and function of microbial communities in a copper bioleaching heap. *BMC Genetics*.

[6] Xiao Y., Xu Y., Dong W. (2015). The complicated substrates enhance the microbial diversity and zinc leaching efficiency in sphalerite bioleaching system. *Applied Microbiology and Biotechnology*.

[7] Hallberg K. B., Johnson D. B. (2001). Biodiversity of acidophilic prokaryotes. *Advances in Applied Microbiology*.

[8] Johnson D. B., Bacelar-Nicolau P., Okibe N., Thomas A., Hallberg K. B. (2009). *Ferrimicrobium acidiphilum* gen. nov., sp. nov. and *Ferrithrix thermotolerans* gen. nov., sp. nov.: heterotrophic, iron-oxidizing, extremely acidophilic actinobacteria. *International Journal of Systematic and Evolutionary Microbiology*.

[9] Watling H. R., Perrot F. A., Shiers D. W. (2008). Comparison of selected characteristics of *Sulfobacillus* species and review of their occurrence in acidic and bioleaching environments. *Hydrometallurgy*.

[10] Segerer A., Langworthy T. A., Stetter K. O. (1988). *Thermoplasma acidophilum* and *Thermoplasma volcanium* sp. nov. from Solfatara fields. *Systematic and Applied Microbiology*.

[11] Johnson D. B., Bridge T. A. M. (2002). Reduction of ferric iron by acidophilic heterotrophic bacteria: evidence for constitutive and inducible enzyme systems in *Acidiphilium* spp.. *Journal of Applied Microbiology*.

[12] Guay R., Silver M. (1975). *Thiobacillus acidophilus* sp. nov.; isolation and some physiological characteristics. *Canadian Journal of Microbiology*.

[13] Johnson D. B., Stallwood B., Kimura S., Hallberg K. B. (2006). Isolation and characterization of *Acidicaldus organivorus*, gen. nov., sp. nov.: a novel sulfur-oxidizing, ferric iron-reducing thermo-acidophilic heterotrophic *Proteobacterium*. *Archives of Microbiology*.

[14] Harrison A. P. (1981). *Acidiphilium cryptum* gen. nov., sp. nov., heterotrophic bacterium from acidic mineral environments. *International Journal of Systematic Bacteriology*.

[15] Hiraishi A., Matsuzawa Y., Kanbe T., Wakao N. (2000). *Acidisphaera rubrifaciens* gen. nov., sp. nov., an aerobic bacteriochlorophyll-containing bacterium isolated from acidic environments. *International Journal of Systematic and Evolutionary Microbiology*.

[16] Azuma Y., Hosoyama A., Matsutani M. (2009). Whole-genome analyses reveal genetic instability of *Acetobacter pasteurianus*. *Nucleic Acids Research*.

[17] Johnson D. B., Roberto F. F. (1997). Heterotrophic acidophiles and their roles in the bioleaching of sulfide minerals. *Biomining: Theory, Microbes and Industrial Processes*.

[18] Li Q., Tian Y., Fu X. (2011). The community dynamics of major bioleaching microorganisms during chalcopyrite leaching under the effect of organics. *Current Microbiology*.

[19] Peng J., Zhang R., Zhang Q., Zhang L., Zhou H. (2008). Screening and characterization of *Acidiphilium* sp. PJH and its role in bioleaching. *Transactions of Nonferrous Metals Society of China*.

[20] Marhual N. P., Pradhan N., Kar R. N., Sukla L. B., Mishra B. K. (2008). Differential bioleaching of copper by mesophilic and moderately thermophilic acidophilic consortium enriched from same copper mine water sample. *Bioresource Technology*.

[21] Thompson J. D., Gibson T. J., Plewniak F., Jeanmougin F., Higgins D. G. (1997). The CLUSTAL_X windows interface: flexible strategies for multiple sequence alignment aided by quality analysis tools. *Nucleic Acids Research*.

[22] Tamura K., Peterson D., Peterson N., Stecher G., Nei M., Kumar S. (2011). MEGA5: molecular evolutionary genetics analysis using maximum likelihood, evolutionary distance, and maximum parsimony methods. *Molecular Biology and Evolution*.

[23] Yin H., Zhang X., Li X. (2014). Whole-genome sequencing reveals novel insights into sulfur oxidation in the extremophile *Acidithiobacillus thiooxidans*. *BMC Microbiology*.

[24] Luo R., Liu B., Xie Y. (2012). SOAPdenovo2: an empirically improved memory-efficient short-read *de novo* assembler. *GigaScience*.

[25] Parks D. H., Imelfort M., Skennerton C. T., Hugenholtz P., Tyson G. W. (2015). CheckM: assessing the quality of microbial genomes recovered from isolates, single cells, and metagenomes. *Genome Research*.

[26] Delcher A. L., Bratke K. A., Powers E. C., Salzberg S. L. (2007). Identifying bacterial genes and endosymbiont DNA with Glimmer. *Bioinformatics*.

[27] Wheeler D. L., Barrett T., Benson D. A. (2007). Database resources of the national center for biotechnology information. *Nucleic Acids Research*.

[28] Kanehisa M., Goto S. (2000). KEGG: kyoto encyclopedia of genes and genomes. *Nucleic Acids Research*.

[29] Altschul S. F., Madden T. L., Schäffer A. A. (1997). Gapped BLAST and PSI-BLAST: a new generation of protein database search programs. *Nucleic Acids Research*.

[30] Finn R. D., Tate J., Mistry J. (2008). The Pfam protein families database. *Nucleic Acids Research*.

[31] Bateman A., Coin L., Durbin R. (2004). The Pfam protein families database. *Nucleic Acids Research*.

[32] Lowe T. M., Eddy S. R. (1997). tRNAscan-SE: a program for improved detection of transfer RNA genes in genomic sequence. *Nucleic Acids Research*.

[33] Lagesen K., Hallin P., Rødland E. A., Stærfeldt H.-H., Rognes T., Ussery D. W. (2007). RNAmmer: consistent and rapid annotation of ribosomal RNA genes. *Nucleic Acids Research*.

[34] Visser J. M., De Jong G. A. H., Robertson L. A., Kuenen J. G. (1997). A novel membrane-bound flavocytochrome c sulfide dehydrogenase from the colourless sulfur bacterium *Thiobacillus* sp. W5. *Archives of Microbiology*.

[35] Wakai S., Tsujita M., Kikumoto M., Manchur M. A., Kanao T., Kamimura K. (2007). Purification and characterization of sulfide:quinone oxidoreductase from an acidophilic iron-oxidizing bacterium, *Acidithiobacillus ferrooxidans*. *Bioscience, Biotechnology and Biochemistry*.

[36] Mangold S., Valdés J., Holmes D. S., Dopson M. (2011). Sulfur metabolism in the extreme acidophile acidithiobacillus caldus. *Frontiers in Microbiology*.

[37] Valdés J., Pedroso I., Quatrini R. (2008). Acidithiobacillus ferrooxidans metabolism: from genome sequence to industrial applications. *BMC Genomics*.

[38] Quatrini R., Appia-Ayme C., Denis Y., Jedlicki E., Holmes D. S., Bonnefoy V. (2009). Extending the models for iron and sulfur oxidation in the extreme acidophile *Acidithiobacillus ferrooxidans*. *BMC Genomics*.

[39] Friedrich C. G., Bardischewsky F., Rother D., Quentmeier A., Fischer J. (2005). Prokaryotic sulfur oxidation. *Current Opinion in Microbiology*.

[40] Friedrich C. G., Rother D., Bardischewsky F., Ouentmeier A., Fischer J. (2001). Oxidation of reduced inorganic sulfur compounds by bacteria: emergence of a common mechanism?. *Applied and Environmental Microbiology*.

[41] Chen L., Ren Y., Lin J., Liu X., Pang X., Lin J. (2012). *Acidithiobacillus caldus* sulfur oxidation model based on transcriptome analysis between the wild type and sulfur oxygenase reductase defective mutant. *PLoS ONE*.

[42] Liljeqvist M., Rzhepishevska O. I., Dopson M. (2013). Gene identification and substrate regulation provide insights into sulfur accumulation during bioleaching with the psychrotolerant acidophile *Acidithiobacillus ferrivorans*. *Applied and Environmental Microbiology*.

[43] Ramírez P., Guiliani N., Valenzuela L., Beard S., Jerez C. A. (2004). Differential protein expression during growth of *Acidithiobacillus ferooxidans* on ferrous iron, sulfur compounds, or metal sulfides. *Applied and Environmental Microbiology*.

[44] Liu L.-J., Stockdreher Y., Koch T. (2014). Thiosulfate transfer mediated by DsrE/TusA homologs from acidothermophilic sulfur-oxidizing archaeon *Metallosphaera cuprina*. *The Journal of Biological Chemistry*.

[45] Stockdreher Y., Venceslau S. S., Josten M., Sahl H.-G., Pereira I. A. C., Dahl C. (2012). Cytoplasmic sulfurtransferases in the purple sulfur bacterium *Allochromatium vinosum*: evidence for sulfur transfer from DsrEFH to DsrC. *PLoS ONE*.

