# Supplementary material for: Genomic Analysis Unravels Reduced Inorganic Sulfur Compound Oxidation of Heterotrophic Acidophilic Acidicaldus sp. Strain DX-1
Source: Biomed Res Int. 2016 Apr 28;2016:8137012. doi: 10.1155/2016/8137012 (PMC4864549; doi:10.1155/2016/8137012)
Supplement: Supplementary file 1 — Table S1: Vairous enzymes associated with RISC oxidation in the Acidicaldus sp. strain DX-1. Table S2: Terminal oxidases involving in the reduced inorganic sulfur compound oxidation of Acidicaldus strain DX-1. [file 8137012.f1.pdf]

Gene	Product	EC no.
hdrA	heterodisulfide reductase subunit A	EC:1.8.98.1
hdrB	heterodisulfide reductase subunit B	EC:1.8.98.1
hdrC	heterodisulfide reductase subunit C	EC:1.8.98.1
dsrE	conserved hypothetical protein	
hdrB2	heterodisulfide reductase subunit B	EC:1.8.98.1
rhd	rhodanese; thiosulfate sulfurtransferase	EC:2.8.1.1
doxD	thiosulfate: quinone oxidoreductase	EC:1.8.5.2
sat2	sulfate adenylyltransferase subunit 2	EC:2.7.7.4
sat1	sulfate adenylyltransferase subunit 1 /adenylylsulfate kinase	EC:2.7.7.4 2.7.1.25
sqr	sulfide quinone reductase	EC:1.8.5.-
sqr2	sulfide quinone reductase, putative	EC:1.8.5.-

Ngar Locus	start	end	AA Length	Best BLAST hits in GenBank database
Scaffold_254_gene_1	1	941	313.333333	*Hyphomicrobium denitrificans* ATCC 51888
Scaffold_254_gene_2	827	2329	501	*Rhodobacter* sp. SW2
Scaffold_254_gene_3	2500	3246	249	*Rhodobacter* sp. SW2
Scaffold_254_gene_4	3322	3702	145	*Rhodobacter sp. SW2*
Scaffold_9_gene_24	22130	22789	220	*Rhodobacter sp. SW2*
Scaffold_153_gene_17	15215	15748	178	*Leptothrix cholodnii* SP-6
Scaffold_68_gene_12	9599	10600	334	*Gluconobacter oxydans* 621H
Scaffold_217_gene_17	14921	15718	266	*Gluconacetobacter diazotrophicus* PAl 5
Scaffold_217_gene_18	16045	16563	173	*Acetobacter aceti* NBRC 14818
Scaffold_18_gene_12	14834	16117	428	*Bradyrhizobium* sp. STM 3809
Scaffold_92_gene_5	4297	5430	378	*Acidithiobacillus ferrooxidans* ATCC 53993
